# Excellent survival after allogeneic HSCT in screened SCID patients and donor type dependent naïve T cell reconstitution

**DOI:** 10.1038/s41409-026-02867-4

**Published:** 2026-05-12

**Authors:** Kajetan Trojovsky, Friedhelm R. Schuster, Hans-Jürgen Laws, Olga Kyrillopoulou, Arndt Borkhardt, Roland Meisel, Sujal Ghosh

**Affiliations:** Division of Pediatric Stem Cell Therapy, Department of Pediatric Oncology, Hematology and Clinical Immunology, Medical Faculty, Heinrich-Heine-University, Duesseldorf, Germany

**Keywords:** Allotransplantation, Haematopoietic stem cells, Innate immunity, Lymphopaenia, Stem-cell research

**To the Editor**

Severe combined immunodeficiency (SCID) is defined by profound numeric or functional T cell deficiency and, without treatment almost all patients die within the first year of life due to severe infections or immune dysregulation [1]. Early and sufficient T cell restoration, typically achieved by allogeneic hematopoietic stem cell transplantation (HSCT), is essential, while gene therapy or enzyme replacement are options for selected entities [2]. Survival has improved to ~90% since implementation of newborn screening [3], yet morbidity remains significant. As adequate total CD4^+^ and naïve CD4^+^ T cell reconstitution is vital [4], we analyzed factors determining immune recovery in a contemporary SCID transplant cohort at the University Children’s Hospital Duesseldorf, Germany, were included in this retrospective study. Data evaluation comprised clinically relevant infections, immune dysregulation, presence of maternal T cells, molecular diagnosis, donor type (haploidentical, MSD/MFD, MUD) and conditioning regimen. Immunological reconstitution was evaluated via flow cytometry of lymphocyte subsets including T (CD3^+^, CD4^+^, CD8^+^, TCRαβ^+^, TCRγδ^+^, CD4^+^ CD45RA^+^), B (CD20^+^) and NK cells (CD56^+^, CD16^+^), chimerism analysis and evaluation of immunoglobulin replacement therapy at defined time points up to 2 years post HSCT. Overall survival was analyzed using the Kaplan-Meier estimator and compared via the log-rank (Mantel-Cox) test. Immune reconstitution data were tested for normality (Shapiro-Wilk/Kolmogorov-Smirnov) and compared using unpaired t-tests or Mann-Whitney U tests (two groups) and ANOVA or Kruskal-Wallis tests (three+ groups). Thirty-nine SCID patients were transplanted between 2000 and 2024. Thirty-five (90%) presented as typical SCID, two as atypical/leaky SCID (*ADA, DCLRE1C*) and two (5%) with Omenn syndrome (both with *RAG1/2*). Twenty-six (67%) had infections or, in ADA deficiency, non-infectious lung disease; 13 (33%) were diagnosed at birth, including 11 by TREC newborn screening which was implemented in Germany in 2019. Genetic causes were identified in 33 patients (85%), including mutations in VDJ recombination (*n* = 16, *RAG1/2*, *DCLRE1C*, *NHEJ1*), T cell receptor signaling (*n* = 9, *IL2RG*, *IL7R*, *JAK3*), *ADA* (*n* = 5), MHC class II deficiency (*n* = 2), and one reticular dysgenesis (Table S1).

The median age at HSCT was 149 days (of note, patients detected by newborn screening were transplanted at a median of 99 days), with most patients receiving haploidentical (*n* = 15) or matched related grafts (MSD/MFD, *n* = 16). Eight received matched unrelated donor (MUD) grafts. 26 received chemotherapy conditioning, 13 patients received graft infusion (infused) without conditioning, 11 of which are MSD/MFD, showing significant difference in conditioning regimes between donor types. 24 patients received anti-thymocyte globulin Grafalon® (ATG) as serotherapy (Supplementary Table S1, S2).

Thirty of 39 patients survived, including all newborn-screened patients (*n* = 13), of whom six received haploidentical TCRαβ/CD19-depleted grafts, whereas unscreened patients had lower survival (65%, *P* = 0.02, Log-rank (Mantel-Cox) test) (Fig. 1A). When stratifying patients to the clinical status at the time of HSCT, 9 of 14 patients with ongoing clinical manifestations died after HSCT, whereas none of the 12 patients with cleared clinical manifestations at HSCT died.

**Fig. 1:**
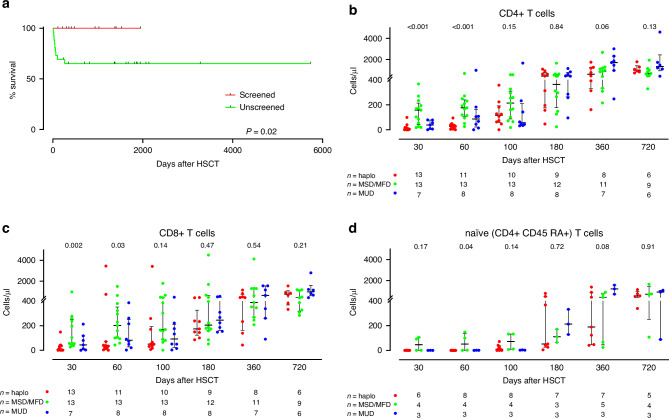
Overall survival and immune reconstitution in SCID. **a** Impact of newborn screening on overall survival (P = 0.02, log-rank, Mantel–Cox-test). **b–d** Impact of donor type (haploidentical vs. MSD/MFD vs. MUD) on CD4^+^ T cell, CD8^+^ T cell and naïve CD4^+^ T cell reconstitution over time. P-values indicate differences between donor groups at the respective time points as assessed by one-way ANOVA or Kruskal–Wallis test, as appropriate. Data are presented as median with interquartile range.

28 of 30 surviving patients achieved adequate immunoglobulin levels within the first year after transplantation and required no ongoing immune replacement therapy.

All surviving patients achieved T cell recovery after HSCT, but kinetics varied by donor type. At day +30 and +60, MSD/MFD recipients exhibited significantly higher CD4⁺ and CD8⁺ (Fig. 1b, c) counts. Median CD4^+^ count at day +60 was 176/µl in MSD/MFD vs. 28/µl in haploidentical and 86/µl MUD recipients, *P* < 0.001, Kruskal-Wallis test, (Fig. 1b).

Naïve CD4^+^ T cell reconstitution also revealed differences between donor groups. At day +60, median CD4^+^ CD45RA^+^ counts were 51/µl (range: 1–148/µl) in the MSD/MFD group, compared to absence of these cell populations in haploidentical and MUD recipients (*P* = 0.04, one-way ANOVA, Fig. 1d). Substantial intra-group variability remained throughout the observation period.

TCRαβ^+^ T cell counts were significantly lower in haploidentical and MUD recipients than in MSD/MFD recipients through day +100, with median counts of 144/µl (range: 1–336/µl) and 135/µl (range: 30–810/µl) versus 347/µl (range: 157–1821/µl), respectively (*P* = 0.02; Kruskal–Wallis test; Supplementary Fig. S1b). In contrast, γδ⁺ T cell counts did not differ significantly between donor types, although a trend toward higher levels was observed in haploidentical recipients at day +60 and +100. (Supplementary Fig. S1c)

Infused patients showed higher rates of mixed chimerism compared to conditioned patients across all time points for total donor chimerism and CD19⁺, CD33⁺, and CD56⁺ lineages. Most importantly, CD3⁺ donor chimerism was similarly high in both groups (Supplementary Fig. S2a).

Similar patterns were observed across graft types, with haploidentical and MUD recipients showing higher CD19⁺, CD33⁺, and CD56⁺ donor chimerism than MSD/MFD, while CD3⁺ chimerism was similarly high among all groups – of patients with available chimerism data, 8 of 10 evaluable MSD/MFD recipients received no myeloablative conditioning (Supplementary Fig. S2b, Supplementary Table S2).

SCID remains a rare, life-threatening disorder of T cell development, requiring HSCT as curative treatment. Our single-center cohort provides insight into early immune reconstitution and outcomes across donor types, highlighting the impact of newborn screening. All screened patients survived, including six receiving TCRαβ/CD19-depleted haploidentical grafts, whereas survival in unscreened patients was 65%, with all deaths occurring within the first year after HSCT due to preexisting morbidity. Survival was particularly poor in patients undergoing haploidentical HSCT in earlier treatment eras. This likely reflects, at least in part, the clinical context in which haploidentical donors were selected, often in patients with significant pre-transplant morbidity where a rapidly available donor represented the only feasible option.

Donor type significantly influenced immune recovery. MSD/MFD grafts showed the most favorable kinetics with earlier CD4^+^ and CD4^+^ CD45RA^+^ reconstitution, especially within the first 60 days after HSCT, while TCRαβ/CD19-depleted haploidentical recipients achieved comparable long-term outcomes despite slower early CD4⁺ and naïve CD4^+^ T cell reconstitution, similar to MUD. TCRγδ⁺ T cell recovery was rapid, whereas TCRαβ⁺ lagged, highlighting the impact of donor selection and graft manipulation [5]. Heterogeneity in conditioning regimens between donor types represents a significant confounder in outcome analyses in our cohort and in other published cohorts [6, 7].

In concordance to other publications, conditioning affected chimerism: infused patients had lower total and lineage-specific donor chimerism except for CD3^+^ [8, 9]. The debate on myeloablative conditioning has recently re-emerged, driven North American data showing no differences in B- and T-cell reconstitution among MSD recipients with or without conditioning [10].

Our findings underscore the critical role of newborn screening in facilitating early, infection-free HSCT, resulting in excellent survival across all donor types. Nevertheless, donor type and conditioning regimen significantly influenced immune reconstitution kinetics and chimerism. Faster naïve CD4^+^ T cell recovery was observed following MSD/MFD transplantation, which may translate into fewer infectious morbidity and reduced hospitalization in larger cohorts. Overall, this study provides detailed insights into early post-transplant immune recovery and supports the development of individualized HSCT strategies for patients with SCID.

## Supplementary information

SUPPLEMENTAL MATERIAL

## Data Availability

The datasets generated and/or analyzed during the current study are not publicly available due to patient confidentiality regulations but are available from the corresponding author on reasonable request and with appropriate institutional approval.
