# Supplementary material for: Excellent survival after allogeneic HSCT in screened SCID patients and donor type dependent naïve T cell reconstitution
Source: Bone Marrow Transplant. 2026 May 12;61(7):951–3. doi: 10.1038/s41409-026-02867-4 (PMC13349852; doi:10.1038/s41409-026-02867-4)
Supplement: Supplementary file 1 — SUPPLEMENTAL MATERIAL [file 41409_2026_2867_MOESM1_ESM.docx]

**Supplementary information**

|  |  | n (total n =39) |
| --- | --- | --- |
| sex | female | 14 |
|  | male | 25 |
| clinical phenotype | SCID | 35 |
|  | atypical SCID | 2 |
|  | Omenn syndrome | 2 |
| genotype | molecular diagnosis established | 33 |
|  | *ADA* | - 5 |
|  | *AK2* | - 1 |
|  | *DCLRE1C* | - 7 |
|  | *IL2RG* | - 5 |
|  | *IL7R* | - 2 |
|  | *JAK3* | - 2 |
|  | *RAG1/2* | - 8 |
|  | *NHEJ1* | - 1 |
|  | MHC class II | - 2 |
| age at HSCT | median 149 days (range 55-2911) |  |
|  | age at HSCT <150 days: | 20 |
|  | age at HSCT >149 days: | 19 |
| graft source | bone marrow | 19 |
|  | peripheral blood stem cells | 18 |
|  | cord blood | 2 |
| donor | matched sibling donor | 11 |
|  | other matched family donor | 5 |
|  | matched unrelated donor | 8 |
|  | - of which are cord blood 8/10 | - 2 |
|  | - of which are 9/10 | - 2 |
|  | mismatched related donor (all haploidentical) | 15 |
|  | - CD34^+^ selected | - 3 |
|  | - CD3^+^/CD19^+^ depleted | - 1 |
|  | - TCRαβ^+^/CD19^+^ depleted | - 11 |
| conditioning | none | 13 |
|  | fludarabine + busulfan | 17 |
|  | other | 9 |
| serotherapy | anti-thymocyte globulin (Grafalon® / Fresenius ®)  none | 24  15 |
| GvHD prophylaxis | mycophenolate mofetil | 10 |
|  | cyclosporine A | 12 |
|  | cyclosporine A + mycophenolate mofetil | 4 |
|  | cyclosporine A + methotrexate | 6 |
|  | none | 7 |
| status at diagnosis | no disease manifestation = screened | 13 |
|  | infection / organ dysfunction / immune dysregulation | 26 |
|  | residual T cells | 18 |
|  | - maternal T cells | - 9 |
| survival | patients alive | 30 |
|  | median day of death: 45 days (range 9-262) |  |

**Supplementary Table S1.** Patient and treatment characteristics

|  |  | Haplo  n=15 | MSD/MFD  n=16 | MUD n=8 |
| --- | --- | --- | --- | --- |
| age at HSCT | median days  (range min-max) | 161 (62-2911) | 131 (55-1217) | 154 (93-417) |
| graft source | bone marrow | 0 | 15 | 4 |
|  | peripheral blood stem cells | 15 | 1 | 2 |
|  | cord blood | 0 | 0 | 2 |
| conditioning | none | 2 | 11 | 0 |
|  | fludarabine + busulfan | 10 | 1 | 6 |
|  | other | 3 | 4 | 2 |
| serotherapy | anti-thymocyte globulin  (Grafalon® / Fresenius ®) | 11 | 5 | 8 |
|  | none | 4 | 11 | 0 |
| GvHD prophylaxis | mycophenolate mofetil | 10 | 0 | 0 |
|  | cyclosporine A | 4 | 6 | 2 |
|  | cyclosporine A + mycophenolate mofetil | 0 | 3 | 1 |
|  | cyclosporine A + methotrexate | 0 | 1 | 5 |
|  | none | 1 | 6 | 0 |
| status at diagnosis | no disease manifestation = screened | 5 | 4 | 4 |
|  | infection / organ dysfunction / immune dysregulation | 10 | 12 | 4 |
|  | residual T cells | 8 | 5 | 5 |
|  | - maternal T cells | 3 | 3 | 3 |
| survival | patients alive | 8 | 14 | 8 |

**Supplementary Table S2.** Cross-tabulation of donor type, conditioning, and serotherapy. Values represent absolute patient numbers.


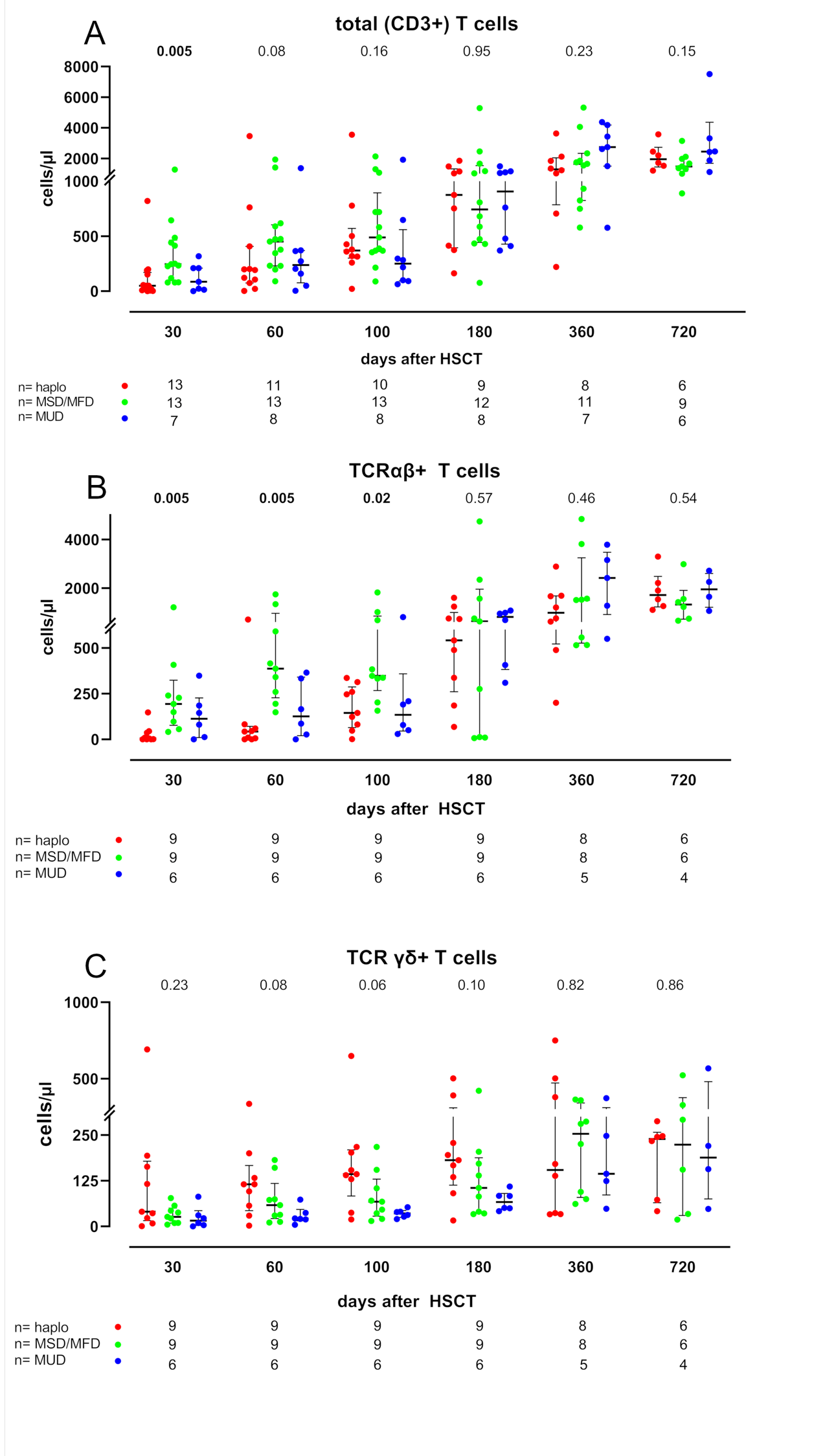


**Supplementary Figure S1:**

Impact of donor type (haploidentical vs. MSD/MFD vs. MUD) on total T (CD3^+^) (A), TCRαβ^+^ (B) and TCRγδ^+^ T cell (C) reconstitution over time. P values indicate differences between donor groups at the respective time points as assessed by one-way ANOVA or Kruskal–Wallis test, as appropriate. The number of evaluable patients per group and time point is indicated below each panel. Panels B and C include fewer patients than panel A because TCRαβ⁺ and TCRγδ⁺ subset analyses were not available for all patients at early time points. Data are presented as median with interquartile range


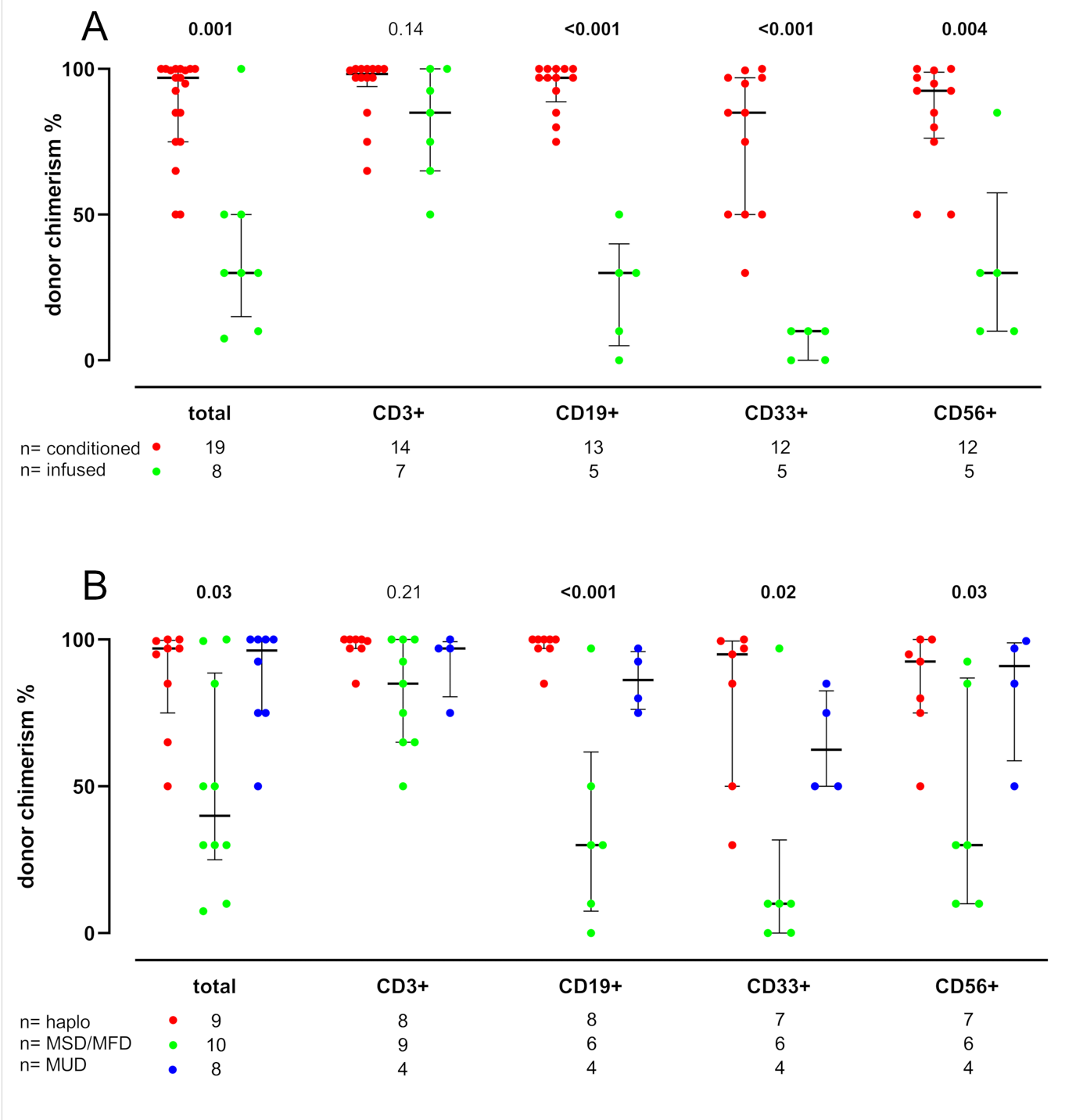


**Supplementary Figure S2:**

Impact of conditioning and donor type on hematopoietic chimerism at day +180 (A) Donor chimerism in conditioned (red) versus infused (green) patients for total leukocytes and indicated subsets (T: CD3⁺, B: CD19⁺, myeloid: CD33⁺, NK: CD56⁺). (B) Donor chimerism by donor type: haploidentical (red), MSD/MFD (green), MUD (blue). Dots represent individuals; bars show median and IQR. Mann–Whitney U test (A) and Kruskal–Wallis test (B). Data are presented as median with interquartile range
